# Oral hypoglycemic agents: do the ends justify the means?

**DOI:** 10.1186/s40748-015-0021-6

**Published:** 2015-08-05

**Authors:** Oded Langer

**Affiliations:** Columbia University, New York, USA

**Keywords:** Gestational diabetes, Glyburide, Metformin, Health research design

## Abstract

**Background:**

Glyburide has replaced insulin as the first line of therapy in the treatment of gestational diabetes in the United States. Glyburide and metformin therapies were reported to be comparable to insulin yet also cost-effective, patient-friendly, and potentially compliance-enhancing. Recently, the efficacy of the use of these oral hypoglycemic drugs has been questioned. In this review, the questionable concerns will be addressed: Which diabetic drug(s) cross the placenta? What is the quality of evidence and the data source validity? Which treatment modalities are most effective in reducing the primary outcome in GDM? Which drug is most effective in improving secondary outcomes?

**Findings:**

This review documents the methodological issues in study design that have impacted the results for the provision of health care interventions in GDM. The review summarizes the contents of the articles qualitatively and assesses the theoretical and empirical evidence. Multiple types of studies exist and every study design serves a specific purpose. Different study designs addressing the same question can yield varying results. The risk of presenting uncertain results without categorically knowing the direction and magnitude of the effect holds true for both randomized and nonrandomized controlled trials. The review further emphasizes the importance of achieving the targeted levels of glycemic control.

**Conclusion:**

The implications of this review are critical to addressing the current gaps in the literature on the efficacy of the use of oral hypoglycemic agents in GDM. The emphasis needs to be placed on patient treatment in order to manage hyperglycemia to reduce fetal and maternal morbidity. In this regard, we need to delineate proper outcome criteria that will reflect disease severity and treat using appropriate pharmacological therapy.

## Introduction

The time has come to place the emphasis on patient treatment of gestational diabetes mellitus (GDM) in order to manage hyperglycemia to reduce fetal and maternal morbidity rather than debating the diagnostic criteria. We need to clearly define outcome criteria that reflect the severity of the disease and the appropriate pharmacological therapy to treat it. Then, randomized and/or other well-designed studies should be performed to demonstrate improvement in perinatal outcome with justified cost of care.

The current American Diabetes Association and European Association for the Study of Diabetes recommendations state that achieving specific glycemic goals can substantially reduce morbidity. Thus, effective treatment of hyperglycemia has become a top priority [1]. Approximately 35-70 % of GDM women will require pharmacological treatment during pregnancy to achieve glycemic goals [2].

Historically, insulin has been the recommended treatment for GDM in the U.S. when dietary and lifestyle measures have failed. Today, randomized controlled trials support the efficacy and short-term safety of glyburide (pregnancy category B) and metformin (pregnancy category B) in the treatment of GDM [3, 4]. The stress free use and low cost of these oral hypoglycemic agents are advantages in comparison to insulin use. These demonstrated advantages have led to a change in clinical practice. Several guideline bodies, including the American College of Obstetrics and Gynecology [5], the UK National Institute for Health and Care Excellence, [6] and the Canadian Diabetes Association [7] endorsed the use of glyburide. Insulin, metformin, and glyburide today are widely used to treat GDM. In general, however, common sense and good clinical judgment should prevail with adjustments for individual preferences, comorbidities, and other patient factors.

In the United States, glyburide use increased from 7.4 % of pharmacological treatment to 64.5 %, superseding insulin as the most common treatment in 2007. Glyburide has replaced insulin as the more common pharmacotherapy for GDM over the past decade [8]. Therefore, it is remarkable that recent studies of glyburide have questioned its use. These studies reported that fetal plasma glyburide concentrations in humans were approximately 70 % of maternal plasma levels [9, 10]. Moreover, 2 observational studies [11, 12] posted warnings about safety issues with the use of glyburide. In this review, several research gaps will be addressed:

1. Which diabetic drug(s) cross the placenta?
2. What is the quality of evidence and data source validity?
3. Which treatment modalities are most effective in reducing the primary outcome in GDM?
4. Which drug is most effective in improving secondary outcomes?

## Findings

### Which diabetic drug(s) cross the placenta?

It is important to note that when measuring placental transfer, one needs to understand the limitations of the measurement system and the unit of measure. 1 mg = 1,000 microgram (μg); 1 μg = 1,000 nanograms (ng); therefore, 1 mg = 1,000,000 ng. Two systems were used to measure placental drug concentration. One is high-performance liquid chromatographic analysis (HPLC-UV) and the mass spectrometry (LC-MS/MS). Comparison of glyburide concentration measured by each system revealed a strong association (y = 0.9321x + 6.9427, r^2^ = 0.9597). However, the level of sensitivity for detection is different in each system.

The majority of drugs used in pregnancy cross the placenta although very few will cause adverse fetal outcome. Transfer of drugs across the placenta is affected by: molecular weight, protein kinase A (pKa), lipid solubility, placental blood flow, blood protein binding, elimination half-life and the specific placental transport system that affects the ability of drugs to enter the fetal compartment.

In glyburide, protein binding is greater than 99 %; it has a short elimination half-life time (6–8 hrs.); the pKa of 5.3 makes glyburide ionize in plasma making it transportable; transport against the concentration gradient from fetus to mother provides a concentration ratio 0.92 ± 0.23; and finally, effluxing glyburide by specific placental transporters. These mechanisms suggest minimal to no transfer of glyburide to the fetal compartment [2].

The recirculating single-cotyledon human placental model is widely used to characterize the transport and metabolism of numerous drugs and nutrients. It has been recognized as an in vitro surrogate for human placental transfer. It facilitates the study of intact human placenta independent of fetal metabolism. Finally, each experiment can be validated with the addition of antipyrine as a reference point for the level of transfer.

Our group [3] demonstrated, using HPLC/UV, that in cord blood samples, minimal glyburide levels (<14 ng/mL) were detectable despite maternal plasma levels of 50-150 ng/ml. Even with increased concentration of glyburide to 100 times the therapeutic level, transport was not appreciably altered. The finding of similar cord-serum insulin concentrations in our two treatment groups also indicates that little, if any, glyburide reached the fetuses. This result confirms in vitro studies [13, 14] in which no maternal-fetal or fetal-maternal transfer of glyburide was detected in full-term placentas perfused immediately after delivery. In addition, glyburide has no effect on the placental transport and uptake of glucose. Furthermore, maternal hyperglycemia does not alter glyburide transfer in vitro. In summary, there was virtually no significant transport of glyburide in either maternal-to-fetal or fetal-to-maternal directions with an average transport of 0.26 % at 2 h. Our findings of negligible glyburide transfer across the placenta were reconfirmed by multiple studies using the single cotyledon model [2].

Two recent studies of glyburide placental transport *in vivo* have caused a furor in the literature [9, 10]. Hebert et al. used LC-MS/MS for the determination of glyburide levels below 10 ng/ml. Maternal blood concentrations ranged from non-detectable to 32.7 ng/ml. The umbilical venous concentrations ranged from non-detectable to12.5 ng/ml. with a mean of approximately 1 ng/ml. Her findings confirmed our original studies of negligible glyburide levels in the fetal compartment.

Hebert et al. [9] reported that the mean maternal-fetal concentration ratio of glyburide was 0.7 ± 0.4. However, concentration is time dependent. Fetal-maternal ratio is the result of a given concentration at a single point in time. Thus, the drug concentration and the ratio are affected by the time of obtaining the sample and the sampling technique used. In our study, [3] the time from administration to obtaining the fetal sample was 8 ± 4 hours with a maternal concentration 50–150 ng. In Hebert’s study [9], the time was up to 13 hours with maternal concentration 0.13-32.7 ng. In Schwartz et al. [10], the time was 13.3 ± 6.5 hours with maternal concentration 15.4 ± 20.8 ng with a range of 0.93-70.71.

Schwartz et al., [10] using LC-MS/MS, found in 15 of the 19 umbilical samples that glyburide concentration was <10 ng/ml. Seven of the 19 umbilical samples showed a higher glyburide concentration than the maternal sample, but only one of these seven measured greater than 10 ng/mL. The overall cord glyburide mean was 7.56 ± 8.2, with a median of 3.7. In summary, all three studies reported negligible levels of glyburide in the fetal compartment. However, the time of sampling differed in each study resulting in different maternal concentrations which, in turn, affects the glyburide concentration ratio between the mother and fetus.

### Does metformin cross the placenta?

Vanky, et al. [15] demonstrated that metformin passes freely across the placenta and fetal serum levels are comparable with maternal values in polycystic ovary (PCO) patients. Tertti, et al. [16] examined in vivo placental transfer of metformin in 217 GDM patients randomized to metformin or insulin. Metformin concentration was determined in maternal serum at 36 weeks gestational age and at delivery. Umbilical cord blood was also obtained at birth. The median umbilical cord/maternal serum metformin concentration ratio was 0.73. Since metformin is a polar positively charged compound, an effort was made to characterize its permeability across the human placenta using the ex vivo placental perfusion model. It was found that metformin permeability across the placenta is mediated by a carrier that transports cationic compounds bi-directionally, with a higher transfer rate from the fetal to the maternal side [17, 18].

### And what about Insulin?

Despite existing dogma that insulin does not cross the placenta, already in the early 80s, it was demonstrated that beef-pork-insulin (antibody) crosses the placenta probably by changing the formation (size) of the antibody insulin complex [19]. More than two decades later, Lindsay et al. [20] reported that insulin antibodies were detected in the cord blood of 95 % of offspring at birth. McCance et al. [21] evaluated the presence of insulin antibodies and their effect on placental transfer with insulin aspart and human insulin. They concluded that insulin antibodies do not develop during pregnancy with the use of aspart in type 1 diabetes and that 1-5 % of human insulin concentration was transferred to the fetal circulation. Boskovic et al. [22] found that no placental transfer could be detected during the perfusion of insulin lispro with 100 and 200 micro U/ml. In contrast, concentration-dependent transfer to the fetus was shown at 580 micro U/ml and higher. Maternal serum level of ≥ 580 U/ml is equal to approximately 75 units of insulin. The authors concluded that insulin lispro is unlikely to cross the placenta at a single standard dose and is also unlikely to reach or harm the unborn baby. Their conclusion was based on the assumption that type 2 and GDM women will probably not receive this amount of insulin. However, this is an erroneous conclusion. GDM and type 2 patients are often overweight and obese, many morbidly obese, characterized by insulin resistance and, therefore, require much larger insulin doses (1–2 units/kg).

In a study of long-acting insulin analogs, the authors suggested that glargine probably does not cross the placenta [23]. Pollex et al. [24] added insulin glargine to the maternal compartment. Experiments were carried out at therapeutic levels 150, 225, and 300 mmol/l. Concentrations of 150pmol/l showed no detectable insulin levels in the fetal compartment. However, after perfusion with concentrations of 150, 225 and 300 nmol/l, the rate of transfer remained low. Thus, transfer begins when the glargine dose is >0.3 unit/kg. The authors concluded that insulin glargine, when used at the therapeutic concentrations, is not likely to cross the placenta. Again, these are flawed conclusions. They are based on the assumption that their designated therapeutic level is universal; it is not. We must all acknowledge that therapeutic levels will be different in different centers related to policy, rate of patients that achieve targeted glycemic goals, obesity, etc.

The view that the placenta is an absolute barrier to drugs is completely inaccurate. The fetus is to some extent exposed to all drugs taken by the mother. There is, however, sufficient evidence to suggest that the placenta is capable of limiting fetal exposure to the drug especially in the case of glyburide. What is important for all drugs, be it oral to insulin, is not which cross the placenta and to which extent, but rather which ones may adversely affect the fetus. To date, thousands of patients have been treated with glyburide, metformin and insulin during pregnancy with no teratogenic effects on the fetus. Patients should not be denied the better treatment option and benefits provided by these drugs; at the same time, future investigations should continue to determine their safety just as they would for any other drug in pregnancy.

### What is the quality of the evidence and data source validity?

Validity is one of the main concerns in research because it applies to both design and methodology. Most errors of validity can be traced to problems with data collection. Therefore, the basic question remains, does the research really measure what it claims to be measuring? Grimes, et al. addressed this major concern in 2 thought-provoking articles [25, 26].

Sources of data abstracted from administrative databases or birth certificates have become expedient and trendy for the potential researcher. However, they are not epidemiologic research data. They are solely the bureaucratic foundation for claims made for services by health care providers and institutions. They are, in fact, billing systems. Actual documentation of events is often related to the probability of reimbursement. These databases were never intended as sources for epidemiologic research because they contain only basic demographic and medical information. They have considerable limitations that must be taken into consideration and should be used solely to abstract information for which they were intended, i.e., billing [8].

For example, an administrative database will rarely, if at all, specify a particular drug (glyburide, metformin, acarbose, etc.) to which the patient was prescribed or exposed but rather will indicate that the patient was prescribed, “an oral agent.” It has been shown that only 59 % of diagnoses in administrative databases were confirmed by actual chart review [27].

Two examples of studies whose data were abstracted from administrative databases demonstrated some of the above limitations. In the study by Castillo, et al. [11], the authors could not ascertain the severity of GDM because of missing data from glucose tolerance tests. The omission of race/ethnicity as well as gestational age in the database precluded the possibility to determine large or small for gestational age infants as potential confounders. They were also limited to the ascertainment of obesity through the use of the International Classification of Diseases (Ninth revision clinical modification) [ICD-9-CM] diagnosis code and could not, therefore, rule out the possibility of the effect of obesity on choice of treatment. In addition, there was failure to provide information on drug dosage. It has become the standard of care that achieving targeted levels of glycemic control is paramount for treatment of GDM women. But, no glucose data during pregnancy was available. A priori, an inability to evaluate the impact of the level of glycemic control during pregnancy should disqualify any study addressing outcome of diabetes in pregnancy Table 1.

**Table 1:** Comparison of Administrative Database Studies

	Castillo et al. [11]			Cheng et al. [12]		
	Glyburide	Insulin	R.R 95 % C.I.	Glyburide	Insulin	O.R. 95 % C.I.
	N = 4982	N = 4191		N = 2073	N = 8604	
LGA	4.7 %	3.2 %	1.43 (1.16-1.76)	17.6 %	19.0 %	1.03 (0.83-1.20)
Macrosomia	n.a.	n.a.	---	13.4 %	13.1 %	1.29 (1.03-1.64)
Hypoglycemia	1.9 %	1.3 %	1.40 (1.00-1.95)	n.a.	n.a.	---
Jaundice	0.3 %	0.4 %	0.96 (0.48-1.91)	n.a.	n.a.	---
Neonatal RDS	2.9 %	1.7 %	1.63 (1.23-2.15)	n.a.	n.a.	---
Preterm	9.5 %	8.9 %	1.06 (0.93-1.00)	n.a.	n.a.	---
NICU admission	10.2 %	7.2 %	1.41 (1.23-1.62)	8.1 %	6.5 %	1.32 (1.07-2.00)
Birth injury	2.2 %	1.6 %	1.35 (1.00-1.82	n.a.	n.a	---
Cesarean delivery	50.6 %	52.5 %	0.97 (0.93-1.00)	38.8 %	44.9 %	0.77 (0.65-0.91)

n.a. = not available, LGA = large for gestational age, NICU = neonatal intensive care unit

Cheng’s, et al. study [12] further exposed the limitations of the administrative database. The authors conducted a retrospective cohort study of women diagnosed with GDM who required pharmaceutical therapy. They were enrolled in the Sweet Success California Diabetes and Pregnancy Program between 2001 and 2004, a California state-wide program. Although they sought to compare insulin and glyburide treated women, the database did not include a variable for a glyburide recipient. Rather, the variable was indicated as “oral agent.” Thus, the authors speculated that all oral agents prescribed were glyburide. The second major limitation of the study was the lack of glucose data throughout pregnancy. Information on neonatal metabolic, respiratory, previous macrosomia or birth trauma data was also not reported Table 1.

The use of administrative databases results in very large sample sizes which, in turn, can lead to spurious statistical associations (“mass significance”). Although the sample size can demonstrate statistical significance, the results may not always be clinically significant. A large sample size, containing confounders, can be challenging when there are weak associations. Risk levels of 1 and 2 should be considered questionable because they may represent potential bias. They are more of a commotion than a signal to consider clinical changes. In general, unless relative risk (RR) in cohort studies exceed 2 to 3 or odds ratios (OR) in case–control studies exceed 3 or 4, associations in observational research findings should not be considered credible. [25, 26, 28] Table 1.

### Which treatment modalities are most effective in reducing primary outcome in GDM?

It has been generally acknowledged that abnormal levels of glycemia result in adverse pregnancy outcome in GDM. Therefore, the achievement of targeted levels of glycemic control is the cornerstone of treatment [1, 29]. Moreover, the majority of studies evaluating the efficacy of glyburide in comparison to insulin used success of achieving glycemic control as the primary outcome variable. Table 2 Appropriately designed randomized controlled trials, or prospective and retrospective studies, with the correct sample size and clear definitions of well and poor glycemic control will maximize production of accurate data [28].

**Table 2:** Achievement of Glycemic Control by Study Design and Treatment Modality

	Design	Patient No. Oral/Insulin	Oral type	Well control Oral / Insulin	Comparison of control
Langer’00 [3]	RCT	201 / 203	glyburide	82 % vs. 88 %	similar
Lain’09 [50]	RCT	41 / 41	glyburide	91 % vs. 97 %	similar
Bertini’05 [51]	RCT	24 / 27	glyburide	79 %	similar
Ogunyemi’07 [52]	RCT	48 / 49	glyburide	93 %	similar
Tempe’13 [53]	RCT	32 / 32	glyburide	91 % vs. 94 %	similar
Silva’07 [54]	RCT	36 / 36	glyburide	---	similar
Rowen’09 [4]	RCT	363 / 370	metformin	54 %	for Metformin
Moore’07 [32]	RCT	31 / 32	metformin	MBG	similar
Spaulonci,’13 [55]	RCT	47 / 47	metformin	74 %	similar
Tertti’13 [16]	RCT	110 / 107	metformin	79 %	similar
Niromanesh’12 [56]	RCT	80 / 80	metformin	86 %	similar
Ijas’11 [57]	RCT	50 / 50	metformin	68 %	similar
Ramos’07 [43]	Retrospective	122	glyburide	84 %	for glyburide
Jacobson’05 [58]	Retrospective	236 /268	glyburide	86 % vs. 63 %	dissimilar
Conway’04 [37]	Retrospective	75	glyburide	84 %	similar
Yogev’10 [38]	Retrospective	124	glyburide	75 %	similar
Rochon’06 [39]	Retrospective	101	glyburide	81 %	similar
Kahn’06 [40]	Retrospective	95	glyburide	81 %	similar

As a rule of thumb, the ability of studies to duplicate a finding is the gold standard of science. Reproducibility of a study is often a rare occurrence. However, regardless of the research design, the majority of studies have consistently demonstrated that insulin and oral agent treated patients (glyburide and/or metformin) achieved the desired level of glycemic control Table 2.

What we don’t yet definitely know is whether metformin or glyburide is the more effective oral hypoglycemic agent. Silva et al., [30, 31] in 2 randomized clinical trials (RCTs) comparing metformin to glyburide, concluded that the achieved levels of glycemia were comparable for both drugs. In addition, there was no difference in perinatal results: cesarean section (C/S), large for gestational age (LGA), neonatal hypoglycemia, or rate of neonatal intensive care unit (NICU) admission [31].

In contrast, Moore et al. [32] in a randomized study showed that the failure rate to achieve the glycemic target was 34.7 % for metformin and 16.2 % for glyburide. In a recent randomized clinical trial (RCT) study, Zohar et al., [33] found that metformin vs. glyburide failed to achieve targeted glycemic levels 29 % vs. 22 %, p = 0.41, respectively. The obstetrical and neonatal outcomes were comparable including anthropometric measures, cord blood insulin and C-peptide. In addition a meta-analysis demonstrated that both metformin and glyburide are as effective as insulin in the management of GDM [34]. The above studies demonstrated comparable success rates and in one a higher failure rate with metformin. However, it is remarkable to note that a meta-analysis [35] reported in favor of metformin for maternal weight gain (mean difference −2.06 kg (−3.98 to −0.14), birth weight (mean difference −209 g (−314 to −104), macrosomia (risk ratio 0.33 (0.13 to 0.81), and large for gestational age newborns (risk ratio 0.44 (0.21 to 0.92), even when treatment failure was higher with metformin. If studies cited are at best inconclusive, meta-analysis simply magnifies the impact of poor quality data.

Disease severity is another factor that influences the success rate in the achievement of targeted glucose levels. The greater the disease severity as reflected by the fasting plasma glucose, success rates to achieve adequate levels of glucose decrease. However, the rates of success remain comparable for glyburide and insulin treated patients at all severity levels [36]. The question remains, how successful are those who failed to achieve glycemic control with either glyburide or metformin after transfer to insulin. The rate of success for achieving glycemic control in metformin treated patients ranged from 54-79 %. The success rate after transfer to insulin was not reported [37–40]. To date, there is no reported data for success of metformin patients transferred to insulin.

The success rate for achieving glycemic control with glyburide ranged from 75-84 %. Transfer failure from glyburide to insulin ranged from 54-67 %. In a logistic regression evaluating predictors of glyburide failure, the main predictor was glyburide dose (O.R. 3.73 95 %, C.I. 2.15-6.48). In addition, there was a weak association (potential bias) for disease severity (O.R. 1.58) and no association to body mass index (BMI), previous GDM, and ethnicity.

Given the approximate doubling in glyburide clearance during pregnancy, should we increase the starting and maximal dose during pregnancy? [9] From the scientific perspective, it is important to understand the pharmacokinetic and pharmacodynamic considerations. However, in the clinical, real world environment, drug management decisions need to be determined based on patient’s diet, weight and ethnicity. In our study, 31 % of GDM subjects achieved targeted levels of glycemic control with a 2.5 mg dose. Twenty-seven percent required 5 mg; 21 % - 10 mg; 9 % - 15 mg; and, only 12 % required the maximal dose of 20 mg [36]. Thus, approximately 2/3 s of the patients achieved targeted levels of glycemic control with less than 50 % of the maximal dose. The amount and frequency of drug administration will both determine the optimal therapeutic effect. The lower limit for glycemic benefits of glyburide are nearly fully realized at half-maximal dose and higher doses should generally be avoided. The upper limit of the range is such that no more than 5-10 % of subjects will experience a toxic effect is a major consideration [41]. In light of this clinical data, it is possible that combination therapy with a lower dose of each medication should be considered in order to successfully maximize glucose control. The goal is to provide drug efficacy without unacceptable toxicity.

### Which drug is most effective in improving secondary outcomes?

A well planned and executed RCT is the gold standard in study design. However, the outcome results that are measured in a single specific trial cannot be regarded as absolute proof. Ioannidis concluded, “Controversies are most common with highly cited nonrandomized studies, but even the most highly cited randomized trials may be challenged and refuted over time, especially small ones” [28].

Different study designs that address the same research question will yield varying results. There is risk of presenting uncertain results for both nonrandomized and randomized controlled trials when there is questionable knowledge if the direction and magnitude of the effect holds true [42]. The majority of maternal and neonatal complications are the result of maternal hyperglycemia that in turn causes fetal hyperinsulinemia. In our RCT we showed that neonatal cord insulin (μU) was similar in glyburide and insulin treated patients (14 ± 4 vs. 15 ± 2.3 respectively) [3]. Ramos et al., in a retrospective study compared glyburide vs. insulin treated GDMs. Neonatal hypoglycemia was defined as <45 mg/dl within 1 h of birth. There was no difference between glyburide and insulin, 23 % vs. 27 %, p = 0.58 [43]. Finally, 2 meta-analyses concluded that neonatal hypoglycemia is not associated with glyburide therapy [44, 45].

### How does glyburide affect the mother?

Yogev et al., [46] using continuous glucose monitoring (CGM) found asymptomatic hypoglycemic episodes in 63 % of insulin and 28 % of glyburide treated subjects. Brustman et al. [47] with a large sample size studied the effect of glyburide therapy on the number of episodes of maternal hypoglycemia in patients using self monitoring blood glucose (mean of 310 ± 190 samples/patient). When hypoglycemia was defined between 40–50 mg/dL, 65 % of subjects had no episodes. When the maternal hypoglycemia definition was <40, over 90 % had no episodes of hypoglycemia. Overall, only 2–3 episodes were found in a minority of patients (~2-4 %).

Several outcome variables are used as secondary outcome measures in the majority, if not all studies, addressing diabetes in pregnancy. These variables are associated with maternal hyperglycemia which in turn causes fetal hyperinsulinemia. Fetal hyperinsulinemia has been shown to be associated with metabolic complications (hyperbilirubinemia, hypoglycemia, respiratory complications, and polycythemia) and the resultant macrosomia/LGA and shoulder dystocia. However, it should be noted that these complications are mainly the result of abnormal glucose levels. In addition, different secondary outcome variables have different weighted importance since some are transitory while others have long term impact on the neonate.

The secondary outcomes in the largest randomized and retrospective studies are compared in Table 3. Within each of the three studies, no significant difference was found in any of the secondary outcome variables. In contrast, the rate of several outcome variables among the three studies was significantly different, (e.g. LGA, macrosomia) probably due to failure to achieve targeted levels of glycemic control. Again, this comparison reemphasizes that the cornerstone for the successful treatment of GDM is the control of the level of glycemia and not the pharmacological treatment modality.

**Table 3:** Summary of Secondary Outcome Results

	Rowen et al. [4]		Langer et al. [3]		Jacobson et al. [58]	
	Randomized		Randomized		Retrospective	
	Metformin	Insulin	Glyburide	Insulin	Glyburide	Insulin
	n = 363	n = 370	n = 201	n = 203	n = 236	n = 268
LGA	19.3 %	18.6 %	12.0 %	13.0 %	25.0 %	24.0 %
Ponderal Index >2.85	---	---	9.0 %	12.0 %	---	---
Macrosomia	22.9 %	21.4 %	7.2 %	4.7 %	25.0 %	24.0 %
Hypoglycemia	15.2 %	18.6 %	9.0 %	6.0 %	31.0 %	27.0 %
Hyperbilirubinemia	8.0 %	8.4 %	6.0 %	4.0 %	25.0 %	22.0 %
Phototherapy	---	---	---	---	9.0 %	5.0 %
Polycythemia	---	---	2.0 %	3.0 %	---	---
RDS	3.3 %	4.3 %	2.0 %	3.0 %	---	---
5 min Apgar	0.8 %	0.3 %	3.1 %	4.2 %	---	---
NICU admission	12.7 %	12.2 %	6.0 %	7.0 %	15.0 %	24.0 %
Shoulder dystocia	---	---	1.5 %	1.6 %	---	---
Preeclampsia	8.3 %	6.8 %	6.0 %	6.0 %	12.0 %	6.0 %
Chronic hypertension	8.5 %	7.3 %	6.0 %	9.1 %	---	---
Cesarean delivery	22.9 %	21.4 %	23.0 %	24.0 %	39.0 %	35.0 %

Systematic reviews and meta-analyses have become increasingly important in health care. Clinicians read them to keep up to date in their field and they are often used as a starting point for developing clinical practice guidelines. Granting agencies may require a systematic review to ensure there is justification for further research. As with all research, the value of a systematic review depends on what was done, what was found, and the clarity of the reported results [48].

Meta-analysis [25, 26, 42] was originally designed for low prevalence conditions to produce hypotheses for future studies. Moreover, including observational studies, in addition to randomized trials, is at best controversial [44, 45]. Several meta-analyses of randomized controlled trials addressing oral agents have been published. However, verification of the methodology and the statistical analysis used in each study are not feasible because the raw data are not available. However, the chance for error(s) is much less in a single study than in a systematic review or meta-analysis. Different designs addressing the same question often yield differences in the results.

For example, in the meta-analysis comparing glyburide to insulin, the largest study contained 404 subjects [3] while each of the other seven studies had fewer than 100 participants for a total of 384 patients Tables 2 and 4. Most of the individual studies were not statistically powered to examine the less common neonatal complications that can be associated with hyperglycemia, such as macrosomia or hypoglycemia. This is of even greater significance when not all seven studies were included in the analysis for each complication. Jiang et al. [34] used 2 studies for the evaluation of cesarean section, and 5 for macrosomia. Zeng et al. [49] reevaluated the rate of neonatal hypoglycemia when only 3 RCTs were included. Euclid, the father of geometry, taught us that the whole is equal to the sum of its parts. However, in meta-analysis, not all parts are equal and very often they are not necessarily included in the whole. A meta-analysis can be no better than its component parts.

**Table 4:** Individual Studies Included in the Various Meta-Analyses

	Dhulkotia [45]	Poolsup [59]	Balsells [35]	Zeng [49]	Jiang [34]	Gui [60]
Metformin studies						
Moore [32]	+	+	+	---	+	+
Rowan [4]	+	+	+	---	+	+
Niromanesh [56]	---	+	+	---	+	+
Ijas [57]	---	+	+	---	+	+
Spaulonci [55]	---	+	+	---	+	---
Tertti [16]	---	+	+	---	+	+
Silva [31]	---	---	---	---	---	---
Hassan [61]	---	---	---	---	+	---
Hague [62]	---	---	---	---	+	---
Therapy efficacy	n.a.	similar	similar	n.a.	similar	similar
Glyburide studies						
Silva [54]	+	+	+	+	+	---
Tempe [53]	---	+	+	+	+	---
Bertini [51]	---	---	---	+	+	---
Ogunyemi [52]	+	+	---	+	+	---
Langer [3]	+	+	+	+	+	---
Lain [50]	---	---	+	+	+	---
Mukhopad-hyay [63]	---	+	+	+	---	---
Therapy efficacy	similar	dissimilar	dissimilar	similar	suitable	n.a.

Table 4 compares 6 meta-analyses with their component studies. The similarities between the studies suggest that there will be comparable results from each meta-analysis. Table 5 compares the outcome in each meta-analysis demonstrating similarity on the one hand and differences on the other. The combination of dissimilar studies can devalue the relevance of any meta-analysis.

**Table 5:** Results of 6 Meta-Analyses Comparing Glyburide to Insulin Therapy

	Dhulkotia 2010 [45]	Poolsup 2014 [59]	Balsells 2015 [35]	Zeng 2014 [49]	Jiang 2015 [34]	Moretti 2008 [44]
Therapy efficacy Glyburide vs. Insulin	similar	dissimilar	dissimilar	similar	suitable	similar
Macrosomia	similar	Increase O.R. 2.34	Increase R.R. 2.62	Increase R.R. 2.22	Increase O.R 3,09	similar
LGA	similar	similar	similar	similar	n.a.	similar
Neonatal hypoglycemia	similar	Increase O.R. 2.06	Increase R.R.2.04	Increase O.R. 1.98	Increase R.R. 2.64	similar
Neonatal hyperbilirubinemia.	n.a.	similar	similar	n.a.	n.a.	n.a
Preterm	n.a.	similar	similar	similar	similar	n.a.
NICU admission	n.a.	similar	similar	similar	similar	similar
Preeclampsia	n.a.	similar	similar	---	similar	n.a.
Cesarean delivery	similar	similar	similar	similar	similar	n.a.

n.a. = not available

In Moretti and Dhulkotia, the meta-analyses included observational and RCT studies in addition to insulin and metformin studies

Outcome definitions and vigorous ascertainment of results were not always provided in the studies. In some outcomes such as preterm birth, caesarean section, birth weight, and Apgar score, there is agreed consensus on definitions. In contrast, in those variables where definitions and detection protocols are less universal, greater differences, i.e., neonatal hypoglycemia, respiratory distress, jaundice, and admission to the neonatal intensive care unit, will result in varying rates in each individual study. For neonatal hypoglycemia, different definitions have been used in the studies with criteria ranging from 45 to less than 25 mg/dl and using from 1–9 blood determinations. In addition, in some studies, administration of I.V. glucose to neonates will classify them as hypoglycemic regardless of the actual blood glucose reading [Tables 4 and 5].

## Summary

The introduction of glyburide and metformin in the treatment of gestational diabetes has profoundly altered the management approach with comparable outcomes to insulin therapy. These therapies are cost-effective, patient-friendly, and potentially compliance-enhancing. Therefore, there may be greater risk to the fetus in withholding these medications than in prescribing them.

The outcome of a clinical debate depends on the adequacy of the respective accounts of existing data. Some types of counter explanations to a concept lack consistency and predictability very often because of faults in validity, i.e. the methodology did not measure what should have been measured. Nevertheless, despite occasional vague explanations, science is better off being tolerant of second-guessers. Science should have both a conservative bias – which prevents rapid and bewildering shifts of views – and eventually openness, such that persistent innovators can ultimately triumph if their claims actually merit consideration. In the words of T.S. Elliot, “Where is the wisdom we have lost in knowledge? Where is the knowledge we have lost in information?”
